# Motivation Peer Training – Bridging the gap for people with mobility disabilities

**DOI:** 10.4102/ajod.v6i0.350

**Published:** 2017-09-08

**Authors:** Lucy K. Norris

**Affiliations:** 1 Programme Development Department, Motivation Charitable Trust, United Kingdom

## Abstract

**Background:**

Only 2% of people with disabilities in developing countries have access to basic services and rehabilitation.

**Objectives:**

To bridge this gap, Motivation has been running Peer Training activities since 1993 and has identified that there is a growing need for Peer Training. The overall aim of Peer Training is for wheelchair users (Peer Trainers) to provide others (with similar disabilities) with the relevant knowledge on health issues, rights and skills to achieve a basic level of independence and greater quality of life.

**Method:**

To test the impact of Peer Training, Motivation created a knowledge, skills and well-being questionnaire, which has been trialled in two locations: Kenya and Malawi.

**Results:**

Overall, Motivation found that most participants reported an increase in knowledge, skills and well-being, supporting their experience that this training provides vital information and support mechanisms for wheelchair users in low- and middle-income countries. Further work is needed to ensure this tool measures the impact of Peer Training and lessons learnt have been identified to strengthen the methodology.

**Conclusion:**

Although Peer Training is not a replacement for rehabilitation services, Motivation believes it is an effective way to not only increase knowledge and skills of persons with disabilities but also reduce the sense of social isolation that can often be a result of disability.

## Introduction

Only 2% of people with disabilities in developing countries have access to basic services and rehabilitation (Despouy 1993). To bridge this gap, Motivation has been running Peer Training activities since 1993 and has identified that there is a growing need for Peer Training. The overall aim of Peer Training is for wheelchair users (Peer Trainers) to provide others (with similar disabilities) with the relevant knowledge on health issues, rights and skills to achieve a basic level of independence and greater quality of life.

To test the impact of Peer Training, Motivation created a knowledge, skills and well-being questionnaire, which has been trialled in two locations: Kenya and Malawi. Overall, Motivation found that most participants reported an increase in knowledge, skills and well-being, supporting their experience that this training provides vital information and support mechanisms for wheelchair users in low- and middle-income countries. Further work is needed to ensure this tool measures the impact of Peer Training and lessons learnt have been identified to strengthen the methodology.

### Background: What is Peer training?

In this context, Peer Training refers to wheelchair users training other wheelchair users with similar mobility disabilities in skills and knowledge that will enable them to carry out everyday activities and achieve an improved quality of life.

#### The history of Motivations’ Peer Training work

Twenty-five years ago, Motivation’s co-founders realised that simply prescribing a wheelchair for someone was not enough to enable independence and increase quality of life. Motivation found that there was very limited knowledge about the specific needs of people with disabilities, especially people with spinal cord injury (SCI). This is suggested by the fact that survival rates remain poor for people with SCI. In low- and middle-income countries, survival rates can be as low as 1–2 years after injury (Gosselin & Coppotelli 2005:330–332). This can often be as a result of fatal infections from untreated pressure ulcers because of the absence of adequate medical care (Gosselin & Coppotelli 2005).

In addition, there is often a social stigma associated with disability which results in marginalisation and isolation (Turk 2009:425–429). This can have a negative psychological impact. For example, 20% – 30% of people with SCI show clinically significant symptoms of depression, substantially higher than the general population (Post & Van Leeuwen 2012:382–389).

In response to this, Motivation has delivered a range of Peer Training activities including one-to-one hospital and home visit sessions and five-day training courses. Initially, Peer Training started out as informal support (i.e. another wheelchair user sharing their experiences). However, recognising that there was a huge gap in rehabilitation for people with disabilities, Motivation decided that a more formal approach was required to reach more people and ensure that good quality training could be delivered consistently in a range of countries.

In 2006, a workshop was held in Tanzania to develop Motivation’s first Peer Training package. Experienced wheelchair users from Kenya, Zimbabwe, South Africa, Uganda, Tanzania and Zambia were invited to review and modify the existing peer resources. The package included a trainer’s guide, posters and handouts focusing on three key areas: disability awareness (e.g. knowledge on SCI and other disabilities, approach to assistants, rights, sexuality and relationships), health (e.g. bladder, bowel and skin care) and mobility (e.g. wheelchair skills, transfers and sports). The package was not targeted at replacing medical professional support, but was created to supplement the support persons with disabilities had already received. To date, 11 500 wheelchair users have participated in Motivation Peer Training (MPT) activities in 22 countries.

In addition to the Peer Training package, a training of trainer’s package was also created to support the development of local Peer Trainers to cascade training. So far, over 100 people have been trained as Peer Trainers, aiming to meet the growing need for Peer Training work.

### Scope

#### The updated package

In 2016, Motivation updated the Peer Training package based on feedback from trainers and trainees in the field through e-mail correspondence and via Skype. The revised MPT package now includes a new participant handbook as well as additional wheelchair skills and sessions on HIV and AIDS and appropriate wheelchairs.

Participants in MPT identified that there needed to be basic information available on the topic of HIV and AIDS but also on the link with disability. This is because people with disabilities are more vulnerable and are frequently forgotten in HIV initiatives. They may be turned away from HIV education forums because of assumptions that they are not sexually active, or do not engage in other risk behaviours (Groce 2004: 1663–1664). There are also physical access barriers for wheelchair users (Opolot 2005). People with disabilities may also feel less empowered to negotiate for safer sex and a large percentage of people with disabilities will experience sexual assault or abuse during their lifetime (American Academy of Pediatrics 2007:1018–1025), which means that people with disabilities are at a greater risk of exposure to HIV.

With this revised package, Motivation hopes to reach more people with disabilities who have limited access to rehabilitation services as it is easier to use and can be accessed upon request.

#### Monitoring the impact of Peer Training

As part of ongoing monitoring and evaluation systems and to meet donor requirements, Motivation’s UK Programme Officer and Africa Peer Training Coordinator developed the knowledge, skills and well-being questionnaire to measure the impact of Peer Training on individual’s lives. So far, the questionnaire has been trialled in two locations: Kenya and Malawi.

#### Ethical considerations

Motivation is a nongovernmental organisation (NGO), not an academic institution. Information gathered was not intended for academic publication but for organisational learning and was therefore not submitted for ethical review. However, informed consent to share anonymised information was sought from all participants involved in the Peer Training in accordance with Motivation’s data and child and vulnerable adult protection policies.

## Key findings

In 2014 and 2015, four MPT courses were run in Malawi, with 13 women and 17 men using the original trial questionnaire – participants either had a SCI or cerebral palsy. In Kenya, the questionnaires were tested during two MPT courses (held in 2016), with 11 women and 12 men – all had SCI, except one person who had spina bifida. Two different versions of the questionnaire were used in Kenya. In the first MPT course, a trial version was used, which was updated based on feedback from trainers and trainees; the updated version was then used in the second MPT course. In both locations, a baseline and an ‘after training’ questionnaire were carried out to analyse the impact of the training (i.e. using average score differentials).

The questionnaire was divided into three sections: knowledge, skills and well-being. The knowledge section looked at three areas: health (e.g. ‘Do you understand the main causes of your disability?’, ‘Do you feel confident in how to care for yourself?’), HIV and AIDS (e.g. ‘Do you know where to receive information, advice and medical treatment on HIV or AIDS?’) and disability rights (e.g. ‘Do you understand your rights as a person with a disability?’).

For this section, it was found that average scores improved from the respondents’ baseline in both Kenya and Malawi, suggesting an improvement in knowledge across all areas. In Kenya, the highest average score increase was for understanding rights; participants listed rights to education, access to public facilities and relationships as a key learning. In contrast, in Malawi, the highest average score change was seen in confidence in caring for themselves.

The mobility skills section of the questionnaire focused on transfers and wheelchair skills such as pushing (e.g. forward/backward/turning), how to get over obstacles and wheelies. In Kenya and Malawi, average score differentials show improvements, specifically in pushing in the community over rough ground. However, other scores such as pushing forward showed little or no change. In addition to the quantitative scoring, qualitative comments demonstrated a change – after the training one participant highlighted ‘wheelchair skills such as obstacles and slopes are valuable – I can now get about more independently’.

The section on well-being looked at a range of factors. In Kenya, average scores improved in most areas, apart from feeling hopeless, which remained the same. More score improvements were seen in Malawi.

The well-being questions on social functioning showed a positive average score change in both Kenya and Malawi. For example, most people suggested they felt less isolated after Peer Training. One of the greatest score improvements was related to people’s perspective on access to recreational activities, as confirmed by comments such as:

‘I feel able to do games and sport.’ (Francis, male, 35 years)

In addition, perceived ability to access religious, political or cultural activities improved; this is substantiated by one person stating:

‘I was scared to go out [before the training]; now I feel confident.’ (Mercy, female, 30 years)

In Malawi, one person stated the training improved her social life, establishing new friendships and invitations to social events (Lewins 2016).

Some of the well-being questions on access to school or work were not applicable to participants. However, those who responded showed an improved average score on whether they felt they could access education or employment. This improvement was supported by the qualitative feedback collected, with one participant stating after the training:

‘I will now attend a school for disability.’ (Marina, female, 23 years)

In Malawi, the external evaluator for this project stated there are successful stories of participants returning to school or some form of education after attending Motivation’s Peer Training (Lewins 2016). One woman stated she was previously unable to live independently but after the training she can go to work and support her family independently (Lewins 2016). Additional examples of people setting up their own businesses were also identified.

## Lessons learned and limitations

At present, the data from the questionnaires are limited because of small sample sizes, modifications, missing data and lack of reliability. It is important to note that although this questionnaire drew on the experience of other validated tests (such as the Wheelchair Skills Test, Dalhousie University 2015), the knowledge, skills and well-being questionnaire was developed for Motivation’s internal use as part of monitoring and evaluation and tailored to meet the needs of users in developing countries.

Motivation has identified areas that may contribute to difficulty in gathering data. For example, Peer Training residential courses are very time sensitive as there are a lot of sessions to cover during a five-day course. Questionnaires can therefore only be carried out when participants arrive and some may need to depart early. Motivation has now changed the questionnaire to be carried out at the end of training. It asks participants to think back to before the training and after the training. Although this approach is not ideal, it should ensure all data are collected and give a general idea of participants’ knowledge, skills and well-being before and after the training.

It must also be recognised that the baseline questionnaire is usually carried out the day before training. Participants may only just have met the Peer Trainer and may not feel comfortable answering questions. In addition, they may feel that they have to give inaccurate responses to please the interviewer. For example, in the Kenya baseline, some participants stated that they felt they had ‘no difficulty’ with some of the wheelchair skills (despite Peer Trainers identifying that not all participants were able to perform some of the skills) – this meant that for some respondents no score change was seen. This demonstrates another flaw in the questionnaire, as participants may feel they are able to do a skill (or try to impress the Peer Trainer), when in fact they may not have been taught to carry it out effectively.

## Conclusion

Motivation’s findings show that, for this sample, there was a positive increase in some domains for people with mobility disabilities. However, the methodology for demonstrating this impact needs further work, based on lessons learned.

It has also been established that while quantifiable evidence is valuable to demonstrate impact of Peer Training, equal value should be placed on qualitative information as this is likely to provide a richer picture of the impact of Peer Training. A participant from Kenya demonstrates this:

‘Overall, Peer Training has improved my quality of life. I am happy now. I will now be able to give back to the community … knowledge is power.’ (Jane, female, 27 years)

Although Peer Training is not a replacement for rehabilitation services, Motivation believes it is an effective way to not only increase knowledge and skills of persons with disabilities but also reduce the sense of social isolation that can often be a result of disability. Motivation will continue to deliver MPT courses to ensure that the needs of people with disabilities are met.

## References

[CIT0001] American Academy of Pediatrics, 2007, *Assessment of maltreatment of children with disabilities, Pediatrics* 119(5), 1018–1025. https://doi.org/10.1542/peds.2007-0565 10.1542/peds.2007-0565 17473105

[CIT0002] Dalhousie University, 2015, *Wheelchair Skills Test 4.3 form for manual wheelchairs operated by their users*, Halifax, Nova.

[CIT0003] Despouy L, 1993, *Human rights and disabled persons*, Study Series 6, Centre for Human Rights, Geneva.

[CIT0004] Gosselin R.A. & Coppotelli C, 2005, ‘A follow-up study of patients with spinal cord injury in Sierra Leone’, *International Orthopaedics* 29, 330–332. https://doi.org/10.1007/s00264-005-0665-3 16094542 10.1007/s00264-005-0665-3 PMC3456651

[CIT0005] Groce N.E. & Trasi R, 2004, Rape of individuals with disability: AIDS and the folk belief of virgin cleansing, *The Lancet* 363, 1663–1664. https://doi.org/10.1016/S0140-6736(04)16288-0 10.1016/S0140-6736(04)16288-0 15158626

[CIT0006] Lewins R, 2016, *Final evaluation on ‘increasing survival and reducing poverty of disabled children and adults in Malawi’ project*. Report unpublished.

[CIT0007] Opolot S.J, 2005, *Challenges faced by people with disabilities in utilizing HIV/AIDS communication and related health services in Uganda*, Action on Disability and Development (ADD), Kampala, Uganda.

[CIT0008] Post M.W.M. & Van Leeuwen C.M.C, 2012, ‘Psychosocial issues in spinal cord injury: A review’, *Spinal Cord* 50, 382–389. https://doi.org/10.1038/sc.2011.182 22270190 10.1038/sc.2011.182

[CIT0009] Turk M.A., 2009, ‘Health, mortality and wellness issues in adults with cerebral palsy’, *Developmental Medicine and Child Neurology* 51: Suppl, 424–29. https://doi.org/10.1111/j.1469-8749.2009.03429.x PMID: 19740207 10.1111/j.1469-8749.2009.03429.x

